# Effects of Creatine Supplementation on Muscle Strength and Optimal Individual Post-Activation Potentiation Time of the Upper Body in Canoeists

**DOI:** 10.3390/nu9111169

**Published:** 2017-10-27

**Authors:** Chia-Chi Wang, Shu-Cheng Lin, Shu-Ching Hsu, Ming-Ta Yang, Kuei-Hui Chan

**Affiliations:** 1 Athletic Department, National Taipei University of Business, Taipei 10051, Taiwan; sunnywang@ntub.edu.tw; 2 Graduate Institute of Athletics and Coaching Science, National Taiwan Sport University, Taoyuan 33301, Taiwan; s0975835@gmail.com (S.-C.L.); jessie800509@gmail.com (S.-C.H.); 3 Center for General Education, Taipei Medical University, Taipei 10031, Taiwan; yangrugby@gmail.com

**Keywords:** complex training, overhead medicine ball throw, bench row

## Abstract

Creatine supplementation reduces the impact of muscle fatigue on post-activation potentiation (PAP) of the lower body, but its effects on the upper body remain unknown. This study examined the effects of creatine supplementation on muscle strength, explosive power, and optimal individual PAP time of the upper body during a set of complex training bouts in canoeists. Seventeen male high school canoeists performed a bench row for one repetition at maximum strength and conducted complex training bouts to determine the optimal individual timing of PAP and distance of overhead medicine ball throw before and after the supplementation. Subjects were assigned to a creatine or placebo group, and later consumed 20 g of creatine or carboxymethyl cellulose per day for six days. After supplementation, the maximal strength in the creatine group significantly increased (*p* < 0.05). The optimal individual PAP time in the creatine group was significantly earlier than the pre-supplementation times (*p* < 0.05). There was no significant change in explosive power for either group. Our findings support the notion that creatine supplementation increases maximal strength and shortens the optimal individual PAP time of the upper body in high school athletes, but has no effect on explosive power. Moreover, it was found that the recovery time between a bench row and an overhead medicine ball throw in a complex training bout is an individual phenomenon.

## 1. Introduction

Using ergogenic aids is a strategy or technique that serves to increase performance during exercise, efficiency of exercise, and recovery after exercise in athletes. Creatine (Cr) is one of the most commonly used nutritional ergogenic aids in various athletic populations and was designed to increase exercise-related strength and power for high intensity exercise bouts of short duration (<30 s) [1]. A number of reviews have reported that combined short-term (five–seven days) Cr supplementation (20 g per day) with exercise/training can significantly increase upper and lower body strength, power, and/or work performance during multiple sets of maximal effort muscle contractions [1,2,3,4].

The neuromuscular phenomenon of post-activation potentiation (PAP) has been applied in training programmes as a complex training and warm-up activity to increase maximal muscle power and strength in athletes, and this application can positively influence long-term training and acute exercise performance. PAP is commonly defined as the enhanced neuromuscular state observed immediately after a session of heavy resistance exercise (HRE) [5,6]. Three physiological mechanisms behind PAP have been purported to contribute to enhance performances after HRE. The proposed mechanisms include increases in the level of neuromuscular activation, phosphorylation of myosin regulatory light chains, and changes in muscle pennation angle [6]. However, studies have indicated both effective [7,8,9,10,11] and ineffective results [10,12,13] on explosive performance after HRE (three–five repetition maximum (RM) strength). Based on the previous research studies and reviews, it is clear that PAP is influenced by muscle fatigue and is an individualized phenomenon [6,10,14,15,16,17].

Previous studies indicated that both muscle fatigue and PAP occur after HRE. Fatigue and PAP have opposing effects on force production and power output in skeletal muscle, hence optimal performance occurs when fatigue has subsided but the potentiated effect still exists [6,14]. Therefore, decreasing muscle fatigue during HRE and recovering faster from muscle fatigue after HRE are important factors for the effectiveness of PAP. Various factors causing fatigue during HRE have been proposed. A decrease in substrate (i.e., adenosine triphosphate (ATP), phosphocreatine (PCr)), an accumulation of metabolic by-products (i.e., lactate, hydrogen ions, and/or inorganic phosphate), and a decreased peak calcium ion concentration in the myoplasm have been associated with fatigue [18]. Previous studies provided compelling evidence suggesting that short-term Cr supplementation (20 g per day for five–seven days) in combination with exercise may augment recovery of skeletal muscle metabolic function and performance [3,19,20,21,22,23,24]. Positive results were observed in our previous study, in which athletes who consumed 20 g of Cr monohydrate for six days could shorten their optimal individual PAP time from 6.13 min to 4.00 min after a 5-RM half squat, but experienced no effect on peak jump performance [25]. Therefore, Cr supplementation has benefits on reducing fatigue after HRE. However, there has only been a small amount of research investigating the effect of Cr supplementation on the upper body [26,27] and no study has evaluated the effect of Cr supplementation on the optimal individual PAP time, strength, and explosive power of the upper body. Thus, the effects of Cr supplementation on the upper body need to investigated.

Several studies have demonstrated a high degree of lower and upper body power after PAP strategies are used in athletes [6,8,9,11,16,28]. However, to date, the majority of PAP studies have usually concentrated on the use of barbell back squats as an effective means for inducing lower body PAP and have investigated effects on lower body performance, such as jump and sprint performance [10,11]. Relatively little emphasis has been placed on the PAP of the upper body. In fact, 94% of PAP studies investigated the effect of PAP on the lower body, as indicated in a review article [6]. The major exercise used in the study of PAP effects on the upper body is the bench press [16]. Moreover, many studies showed that recovery time between HRE and a subsequent explosive activity should be individualized, because athletes’ backgrounds (including muscle fibre type, training experience, and strength level) and the workout structure (type of conditioning activity, intensity of HRE) would affect the PAP response [6,14]. Studies have suggested that the optimal recovery period may vary, including values of 5 min [9], 8 min [16,29], 4–12 min [30], 7–10 min [14], and 8–16 min [31]. However, these studies used the half squat or bench press as the HRE. The bench row is a multi-joint resistance training exercise commonly used in sport disciplines that require upper body pulling, such as canoeing, rowing, and kayaking. A study using different types of exercise to fit different events is necessary to apply the concept of PAP to the upper body.

To the best of the author’s knowledge, the effects of Cr supplementation on increasing PAP effects on the upper body have not been investigated. Moreover, the bench row exercise, a common method of upper body training, has not been considered in this context. Therefore, the goal of this study was to examine whether short-term Cr supplementation can attenuate the impact of fatigue on muscle power performance and effectiveness of the optimal individual PAP time between the bench row and a subsequent explosive activity, as well as whether this supplementation can increase upper body strength. It was hypothesized that Cr supplementation would increase upper body strength, increase power performance, and shorten the optimal individual PAP time during a complex training bout, and that the recovery time of the upper body during a complex training movement (bench row and medicine ball throw) would be an individual phenomenon.

## 2. Experimental Section

### 2.1. Research Design

To examine the upper body results, the study procedure and method were similar to those of our previous study [25]. Before formal measurements, all subjects visited the laboratory initially to ensure familiarity with the bench row and overhead medicine ball throw (OMBT) technique. Subjects were educated during the familiarization session by a well-trained fitness instructor. The day after the familiarization session, anthropometric indexes and the strength of a one repetition maximum (1-RM) bench row were measured. Two days later, subjects performed two sets of complex training bouts with six 2-min rest intervals by two separated days to determine the individual optimal timing of PAP, and the distance of an OMBT. A double-blind, randomized design was used to assign 17 subjects into a Cr group or a placebo (Pla) group. After six days of high dose Cr or Pla supplementation, the same test procedures performed before supplementation were conducted again to evaluate the effects of Cr supplementation. A low dose of Cr or Pla supplementation was maintained until the end of the study. All familiarization and experimental sessions of this study were performed at the same time (from 10 AM to 2 PM) each day. The study was approved by the Institutional Review Board of the Fu Jen Catholic University, Taiwan.

### 2.2. Subjects

Seventeen male high school canoeists volunteered to participate in this study. The characteristics of the subjects are described in Table 1. All subjects provided written informed consent before participation. They maintained their basic training programmes and were asked to keep their normal dietary patterns during the experimental period. Subjects were excluded if they had one of the following: (1) injury to an upper limb within the past six months; (2) experience with bench row and OMBT training within the past six months; or (3) use of chronic or daily doses of anti-inflammatory medications or nutritional supplements within the past month.

### 2.3. Supplementation Protocol

After the baseline testing, subjects in the Cr group began consuming 5 g of pure unflavored creatine monohydrate powder (creatine fuel powder; Twinlab, Hauppauge, NY, USA) plus 5 g of dextrose dissolved in 300 mL of water four times (at breakfast, lunch, dinner, and before bedtime) per day for six days. Subjects in the Pla group followed the same protocol but consumed carboxymethyl cellulose (food grade CMC powder, GreenYoung Co., Taichung, Taiwan) instead of Cr. The supplements for both groups were the same colour and taste. For maintenance, subjects ingested single daily doses of 2 g of creatine monohydrate or carboxymethyl cellulose powder plus 2 g dextrose dissolved in 200 mL of water after lunch until the end of the study.

### 2.4. Prediction of One Repetition Maximum Strength

Prediction of 1-RM strength for the bench row was determined based on the protocol described by Baechle et al. [32]. In brief, subjects jogged for 5 min on a treadmill followed by lower/upper limb light stretching exercises and two light resistance warm-up sets. After 1 min of rest, the subjects were instructed to lie prone on the high bench (Apex B45 adjustable flat bench) and hold a barbell at a load of 87−93% of the predicted 1-RM. On command, the subject raised the bar to the bottom of the bench and then lowered the bar back to full elbow extension. After each successful performance, the load was increased in increments of 8–10% until only one successful repetition could be completed. Four minutes of rest were given between each test. The increase or decrease in the load continued until the subject was able to complete one repetition with the proper exercise technique. Ideally, the subject’s 1-RM was measured within five testing sets.

### 2.5. Optimal Individual PAP Time and Overhead Medicine Ball Throw Test

The OMBT test was selected to evaluate upper body muscular power. Studies have shown that the OMBT test is a valid and reliable test for assessing upper body muscular power and is commonly used for testing upper body power [33,34,35]. After a low intensity aerobic exercise followed by a light stretching exercise for warm-up, subjects performed two OMBT tests for baseline measurements. During the OMBT test, subjects stood at a line with feet slightly apart, and a 3-kg medicine ball was brought back behind the head, then subjects threw the medicine ball as far forward as possible. The subjects were not allowed to move their feet during the test. Each OMBT was separated by a 5-s rest period. The longest OMBT value was used in the analysis.

After a 5-min rest, subjects executed a set of complex training bouts involving 3-RM bench row exercises to elicit PAP followed by a counterbalanced order of six rest intervals (1, 3, 5, 7, 9, 11 min or 2, 4, 6, 8, 10, 12 min) for two days. The optimal individual PAP time was the rest interval with the maximum delta-values for the throw distance during the complex training bouts minus the baseline values.

### 2.6. Anthropometric Measurements

All subjects visited the laboratory in the morning for anthropometric measurements including body height (cm), body mass (kg), and body fat percentage (%). Standing body height without shoes or socks was measured to the nearest 0.1 cm with a height meter mounted on a wall. Body mass and body fat percentage were measured by a bioelectrical impedance instrument (InBody 3.0, Biospace, Seoul, Korea) with standard methods used to assess body composition.

### 2.7. Statistical Analysis

Statistical analyses were performed using SPSS version 19.0 software (SPSS Inc., Chicago, IL, USA). Data are expressed as the means ± standard deviation. An independent sample *t*-test was used to compare the subjects’ characteristics between the groups. A mixed design two-way ANOVA (group × time) was used to compare the variables of 1-RM strength, OMBT distance, and optimal individual PAP time. Statistical significance was set at *p* < 0.05.

## 3. Results

### 3.1. Subject Characteristics

Subject characteristics for both groups are presented in Table 1. No significant differences were noted for any variable (*p* > 0.05).

### 3.2. Effects of Cr Supplementation on Maximum Upper Body Muscle Strength and Explosive Power in a Set of Complex Training Bouts

**Figure 1:**
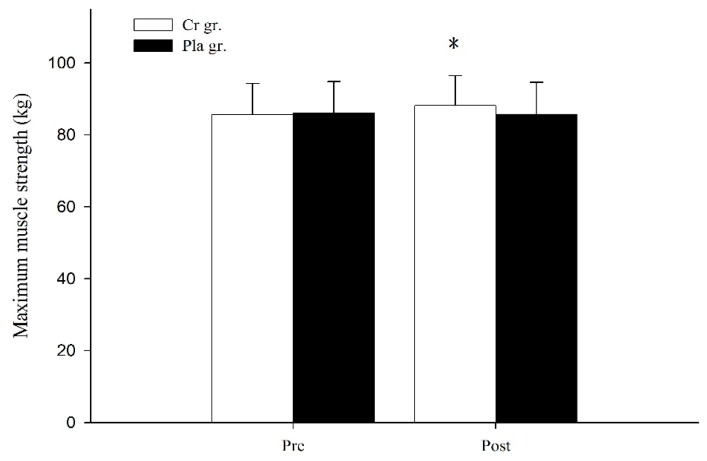
Maximum muscle strength of bench row before and after six days of creatine or placebo supplementation. Data are the means ± standard deviation. Cr gr. = creatine group; Pla gr. = placebo group; Pre = pre-supplementation; Post = post-supplementation. Asterisk (*) indicates a significant difference (*p* < 0.05) from the pre-supplementation value within the group.

**Figure 2:**
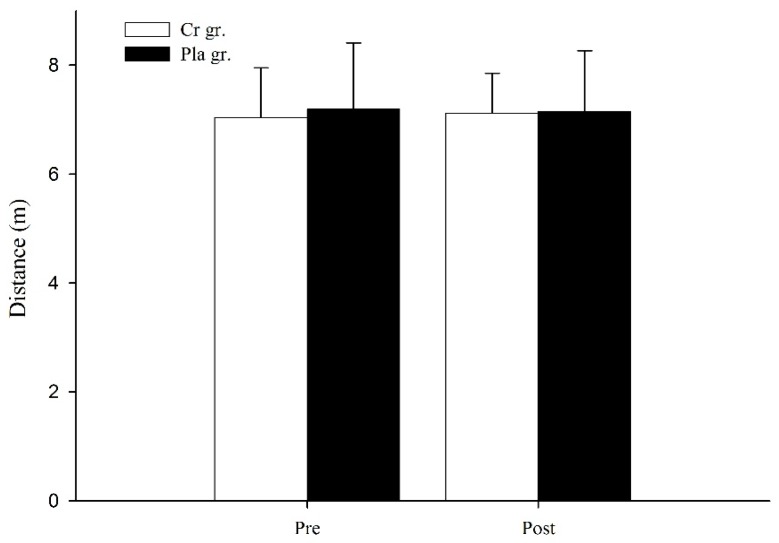
Distance of overhead medicine ball throw after the optimal individual post-activation potentiation (PAP) time in complex training bouts before and after six days of creatine or placebo supplementation. Data are the means ± standard deviation. Cr gr. = creatine group; Pla gr. = placebo group. Pre = pre-supplementation; Post = post-supplementation.

Figure 1 shows the results of a 1-RM strength bench row before and after six days of Cr or Pla supplementation. There was a significant interaction between groups and time. Following supplementation, 1-RM strength in the Cr group significantly increased from 85.63 ± 8.63 kg to 88.12 ± 8.36 kg (*p* < 0.05). However, there were no significant differences in the Pla group or between the Cr and Pla groups (*p* > 0.05). There was no significant change in OMBT distance after the optimal individual PAP time in complex training bouts for either group (*p* > 0.05, Figure 2).

### 3.3. Optimal Individual PAP Time

**Figure 3:**
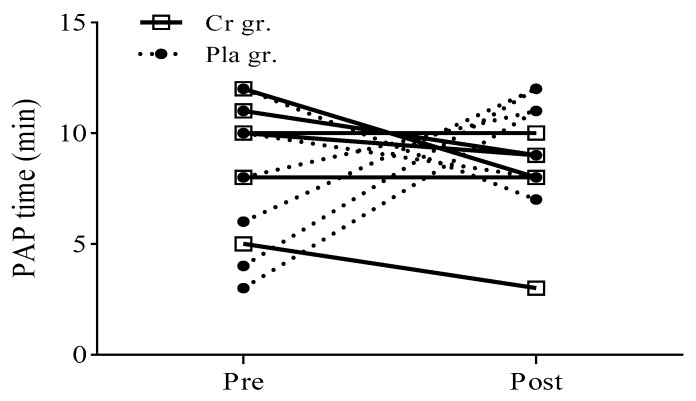
Optimal individual PAP time for each subject following creatine or placebo supplementation. Cr gr. = creatine group; Pla gr. = placebo group; Pre = pre-supplementation; Post = post-supplementation.

Figure 3 shows the optimal individual PAP time points for each individual and illustrates the individual variations in the results. The two groups had their optimal PAP times at different time points. Furthermore, there was a significant interaction between groups and time. After supplementation, the optimal individual PAP time in the Cr group was significantly earlier than it was in that group pre-supplementation from 9.75 ± 2.31 min to 8.12 ± 2.23 min (*p* < 0.05). However, there were no significant differences in the Pla group or between the Cr and Pla groups (*p* > 0.05).

## 4. Discussion

The present study is the first to assess the potential efficacy of short-term Cr supplementation in improving upper body performance in a complex training bout in canoeists. The major findings of this study are that Cr supplementation significantly increased the maximal strength of the bench row and reduced the negative influence of fatigue on the optimal individual PAP time during a set of complex training bouts involving the upper body (3-RM bench row and overhead medicine ball throw). However, this acute benefit could not enhance explosive power during a set of complex training bouts.

The primary and original results of this study indicate that the optimal individual time required to maximize the effect of PAP on the upper body was significantly earlier after Cr supplementation, decreasing from 9.75 min to 8.12 min after supplementation. Based on previous studies, explosive muscle contractions depend on the balance between fatigue and PAP, and fatigue is more dominant in the early stage of recovery [36]. Therefore, it is possible that Cr supplementation caused less fatigue, thereby reducing the diminishing effect of fatigue on the recovery period and allowing the PAP effect to predominate in the early recovery period. Another proposed reason for this result is that Cr supplementation facilitates the reuptake of calcium ions into the sarcoplasmic reticulum [37], thus activating more phosphorylation of myosin regulatory light chains, which is one of the principal mechanisms of PAP [6]. This result was consistent with our previous study [25], in which we observed that Cr supplementation for six days shortened the optimal individual PAP time from 6.13 min to 4.00 min after a 5-RM half squat. In addition, our findings are in agreement with previous studies showing that five days of Cr supplementation (20 g/day) significantly increased the total repetitions performed before fatigue and the total average power output values during repetitive high-power-output exercise bouts involving the upper body [3,23], and increased the time to fatigue during three bouts of submaximal knee extension and isometric handgrip [21]. In theory, Cr supplementation can delay the onset of fatigue during anaerobic exercise by decreasing the contribution of anaerobic glycolysis and then reducing lactate and hydrogen ions accumulation [1]. A meta-analysis revealed the effect of Cr supplementation on performance improvement in high-intensity exercise lasting ≤30 s [19]. The present findings suggest that the mechanisms of Cr work to decrease muscle fatigue by increasing the intramuscular concentration of PCr, aiding the rephosphorylation of adenosine diphosphate (ADP) to ATP, reducing pH changes from acidosis by using the hydrogen ions during the creatine kinase reaction and stimulating phosphofructokinase activity. Therefore, there is evidence to support our hypothesis that short-term Cr supplementation has a benefit in attenuating muscle fatigue symptoms and increasing recovery after HRE of the upper body. In addition, our results support previous studies that indicate that the PAP phenomenon is highly individualized. The subjects of our study had their highest PAP performance of complex training involving the upper body within a broad range of rest intervals (3, 4, 5, 6, 8, 10, 11, 12 min), which was consistent with our previous finding [25] that some subjects had the greatest PAP effect at 3–6 min after HRE, whereas this time interval varied for other subjects. Similar findings were observed in studies by Naclerio et al. [38] and Conmyns et al. [15] that indicated that the participants’ best performances occurred between 15 s and 12 min. These studies concluded that this result could be attributed to the participants’ differing background factors, including training level, strength level, and training experiences. Therefore, the obtained result supports our hypothesis that the optimal individual PAP time for a complex training set consisting of a bench row and medicine ball throw would also be influenced by the individual’s background.

Although the optimal individual PAP time was significantly earlier after Cr supplementation, decreasing from 9.75 min to 8.12 min, we observed that the elicited PAP was not sufficient to enhance the peak performance in the OMBT test. This observation is in line with our previous finding, which showed that Cr supplementation did not significantly improve the performance of countermovement jump, despite the fact that the optimal individual PAP time was earlier after supplementation [25]. This result may be explained by the gradual rate of PAP. Previous studies have indicated that the peak PAP is achieved immediately after HRE, but instantly begins to decrease for the remainder of the recovery period. Several studies have assessed the time course of PAP decline after maximal voluntary contraction (MVC) of knee extensors [39,40,41,42,43,44]. Pääsuke et al. [41] showed that peak twitch was potentiated immediately by 51% after a single 10-s MVC of knee extensors, and there was a sharp decline in potentiation during the first 3 min of recovery, although potentiation was still higher than the pre-MVC value at 5–10 min. This magnitude and time course of PAP decline was similar to those observed in another study, in which twitch potentiation was induced in the knee extensor muscles by a 10-s MVC; the PAP of the twitch peak torque increased by 70.6% immediately but then rapidly declined to +31% at 60 s. Potentiation becomes more gradual over time, resembling an exponential function [39]. Hamada et al. [43] also indicated a greater decline in torque during a 10-s MVC in the elbow extensor compared to the ankle plantarflexor muscles. Similarly, Seize et al. [44] indicated that 6-s maximal dynamic knee extensions elicited significantly increased PAP from 1 to 7 min, and potentiation was still higher than the pre-test value at 7–13 min. Another study also concluded that twitch potentiation in the knee muscles was potentiated within 3–10 min of recovery [42]. Therefore, although Cr supplementation shortened the optimal individual PAP times in our study, the elicited PAP was not sufficient to enhance explosive power, because the peak PAP value had already elapsed.

Additionally, the findings of this study indicated that supplementation with 20 g/day of Cr for six days increased the maximal strength of a bench row. This present finding was consistent with previous studies [2,3,4], systematic reviews [1], and a meta-analysis [27]. These studies concluded that the potential mechanisms of the acute effect of short-term Cr supplementation (20 g/day for five–seven days) on the maximal upper body strength involves an increase in intramuscular PCr stores, allowing for rapid rephosphorylation of ADP back to ATP to delay the onset of muscular fatigue and improve Ca^2+^ kinetics in the sarcoplasmic reticulum.

## 5. Conclusions

This study suggests that short-term Cr supplementation in male high school canoeists resulted in improved upper body maximum strength and shortened optimal individual PAP times for training efficiency during a set of complex training bouts involving the upper body. Although short-term Cr supplementation is not enough to improve the explosive power of the upper body, it appears to be an effective supplementation method with respect to efficiency and strength development. Conditioning coaches may apply the results of this study to design proper complex training programs to enhance the performance of specific sports. Moreover, our results support the idea that the PAP phenomenon after a 3-RM bench row is also highly individualized. Further studies could apply the supplement strategy to investigate the effects on the efficiency of long-term complex training involving the upper body and/or lower body for the performance of specific sports.

## Figures and Tables

**Table 1:** Subject characteristics.

Variable	Cr group (*n* = 8)	Pla group (*n* = 9)
Age (years)	16.75 ± 0.70	16.44 ± 1.13
Height (cm)	169.48 ± 3.61	172.16 ± 3.53
Weight (kg)	65.33 ± 4.65	64.34 ± 7.14
Body fat (%)	14.50 ± 2.58	13.20 ± 2.96

Data are the means ± standard deviation. Cr = creatine; Pla = placebo.
